# The Therapeutic Effects of *Nigella sativa* on Skin Disease: A Systematic Review and Meta-Analysis of Randomized Controlled Trials

**DOI:** 10.1155/2022/7993579

**Published:** 2022-12-05

**Authors:** Naser Nasiri, Mozhde Ilaghi Nezhad, Fariba Sharififar, Mahdieh Khazaneha, Mohammad Javad Najafzadeh, Neda Mohamadi

**Affiliations:** ^1^HIV/STI Surveillance Research Center, and WHO Collaborating Center for HIV Surveillance, Institute for Futures Studies in Health, Kerman University of Medical Sciences, Kerman, Iran; ^2^Leishmaniasis Research Center, Kerman University of Medical Sciences, Kerman, Iran; ^3^Herbal and Traditional Medicines Research Center, Kerman University of Medical Sciences, Kerman, Iran; ^4^Neurology Research Center, Kerman University of Medical Sciences, Kerman, Iran; ^5^Student Research Committee, Kerman University of Medical Sciences, Kerman, Iran

## Abstract

The aim of this systematic review was to identify randomized controlled trials that looked at the effects of *Nigella sativa* in any form on different skin diseases. Up to March 2022, the online databases of Scopus, Web of Science, PubMed, Embase, Google Scholar, and Cochrane trials were searched. This study included 14 records of people who had experienced different types of skin disease including atopic dermatitis, vulgaris, arsenical keratosis, psoriasis, vitiligo, acute cutaneous leishmaniasis, warts, eczema, and acne. The mean SD age of the patients was 28.86 (4.49); [range: 18.3–51.4], with females accounting for 69% (506 out of 732) of the total. The follow-up mean SD was 8.16 (1.3) (ranged: 4 days to 24 weeks). The odds ratio (OR) was found to be 4.59 in a meta-analysis (95% CI: 2.02, 10.39). Whereas the null hypothesis in this systematic review was that lotion had no impact, OR 4.59 indicated that lotion could be effective. The efficacy of *N. sativa* essential oil and extract has been demonstrated in most clinical studies. However, more research is needed to completely evaluate and validate the efficacy or inadequacy of therapy with *N. sativa*, although it appears that it can be used as an alternative treatment to help people cope with skin problems.

## 1. Introduction

The skin is the largest organ and functions as a barrier to protect the underlying tissues against the elements and pathogens, while also fulfilling many physiological roles and biochemical functions such as preventing excessive water loss [1]. Skin diseases have recently become a major concern among people of all ages due to their highly visible symptoms and persistent and difficult treatment that have a significant effect on quality of life [2].

*Nigella sativa* belongs to the Ranunculaceae family is an annual plant which distributed in southern Europe and some parts of Asia, including Syria, Turkey, Saudi Arabia, Pakistan, and India. Different active pharmaceutical ingredients have been identified in the *N. sativa* seeds, including saponins, flavonoids, cardiac glycosides, thymoquinone, thymol, limonene, carvacrol, p-cymene, alpha-pinene, 4-terpineol, longifolene, t-anethole benzene, isoquinoline, and pyrazole alkaloids, as well as unsaturated fatty acid such as linoleic acid, oleic acid, and palmitic acid [3]. Food and therapeutic uses of *N. sativa* oil seeds have a long history in Persian traditional medicine. Avicenna, in his famous book, The Canon of Medicine, has reported several black cumin properties, such as fatigue improvement and energy recovery. It has been traditionally used for the treatment of asthma, bronchitis, and rheumatism.

Animal models have shown the therapeutic effects of *N. sativa* on acne vulgaris, burns, wounds, and injury [4–7], skin inflammation [8], and skin pigmentation [9].

Since traditional treatments have become widely popular in recent decades, it is imperative to provide patients with skin diseases enough evidence-based alternatives to help them manage their symptoms. The aim of this systematic review and meta-analysis was to evaluate the overall effectiveness of *N. sativa* products for treating skin problems.

## 2. Methods

In this systematic review and meta-analysis, we preferred reporting items according (PRISMA) guideline (Supplementary file S1).

### 2.1. Data Sources

The electronic databases, including PubMed, Scopus, ISI Web of Science, Cochrane Central Register of Controlled Trials (CENTRAL), and Google Scholar (Supplementary file S2) were searched until March 2022. To find more relevant studies, the reference lists of all eligible studies and previous reviews were reviewed manually.

### 2.2. Search Strategy and Study Selection

The MESH and non-MESH search terms applied were (“*Nigella* *sativa*^*∗*^” OR “Kalonj^*∗*^” OR “Black Cumin^*∗*^”) AND (“Acne Vulgaris” OR Dandruff OR “Atopic Dermatitis” OR “Contact Dermatitis” OR “Exfoliative Dermatitis” OR “Perioral Dermatitis” OR “Seborrheic Dermatitis” OR Eczema OR Hirsutism OR Ichthyosis OR “Seborrheic Keratosis” OR “Cutaneous Lupus Erythematosus” OR “Discoid Lupus Erythematosus” OR “Phototoxic Dermatitis” OR “Phototoxic Dermatitis” OR “Hyperpigmentation” OR “Hypopigmentation” OR “Pruritus Ani” OR “Pruritus Vulvae” OR “Acne Vulgaris” OR “Seborrheic Dermatitis” OR “Psoriasis”). In our search strategy, study designs, participants, publication time, and language were deliberately not limited in order to facilitate finding all the relevant studies. All searches were conducted by two researchers (NM and MI) independently. Duplicated studies were then eliminated. In general, these two authors had an agreement on selecting the studies, and possible variations were removed by discussion.

### 2.3. Inclusion and Exclusion Criteria

If a study met the following criteria, it was considered for inclusion: (1) patients with mild to severe skin disorders were recruited, (2) using of any products of *N. sativa* in the forms of oral and topical (3) the type of skin disorders conditions experienced by the participants was not restricted, (4) utilized *N. sativa* in combination with other plants, phytochemicals, drugs, or supplements; (5) controlled clinical trials of either parallel or crossover design.

We excluded research that involved: (1) Publications without accessible English abstracts, as were reviews, case reports, comments, theses, book sections, and conference proceedings. (2) Employed animal models.

### 2.4. Data Collection

Two authors (NM and MI) independently extracted data, including first author, publication date, study type, location, total, sample size, age, sex, and dose, form (powder, oil, and extract), duration of treatment, main outcomes (mean and standard deviation), and adverse effects.

### 2.5. Quality assessment of the Evidence

The Joanna Briggs Institute critical appraisal technique was used to assess the quality of the studies included. Joanna Briggs proposed 13 criteria for evaluating the quality of randomization clinical trial trials. The Joanna Briggs Institute critical evaluation was also used for case series and quasi-experimental investigations. Adjudication was utilized to resolve differences in the grade of risk of bias studies.

### 2.6. Statistical Analysis

Data were presented using descriptive statistics (mean ± SD) for continuous variables; frequency and percentage for categorical variables. We applied random-effectsmeta-analysis to calculate risk ratio and 95% confidence interval in Stata version 14.2.

## 3. Results

### 3.1. Studies Characteristics

**Figure 1:**
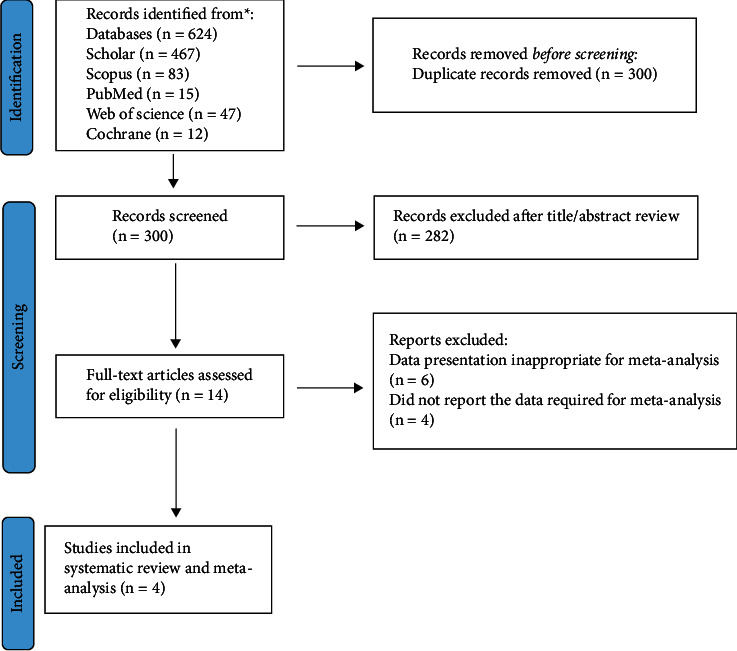
Flowchart of studies included in the systematic review the therapeutic effects of *N. sativa* on skin disease.

The list of included studies on skin disease therapeutic effects with *N. sativa* is shown in Table 1. In all, 14 records out of 300 unique articles were possibly eligible; ultimately, 4 papers were included in this meta-analysis [11, 12, 14, 15] (Figure 1). Of the 14 included studies, one was conducted in Germany [10], the Czech Republic [11], Tukey [22], India [13], and Bangladesh [17], two were conducted in Iraq [14, 18], and the other was carried out in Iran [12, 15, 16, 19–21, 23]. Studies were done on individuals who had experienced different types of skin disease, as listed in Table 1. In the clinical trials studied in this review, *N. sativa* oil was administered in 12 studies, and in two *N. sativa* studies crude extract was administered [12, 18].

### 3.2. Adverse Effects

Out of the three studies that evaluated the adverse effects of treatment with *N. sativa*, Kalus et al. reported transient gastrointestinal problems [11]. One study reported that 62% of participants in the invention group had gastric irritation, including abdominal cramps, and indigestion [17], and the other 5 out of 75 patients in the *N. sativa* group (6.7%) reported topical side effects among patients [15].

### 3.3. Findings from the Meta-Analysis

**Figure 2:**
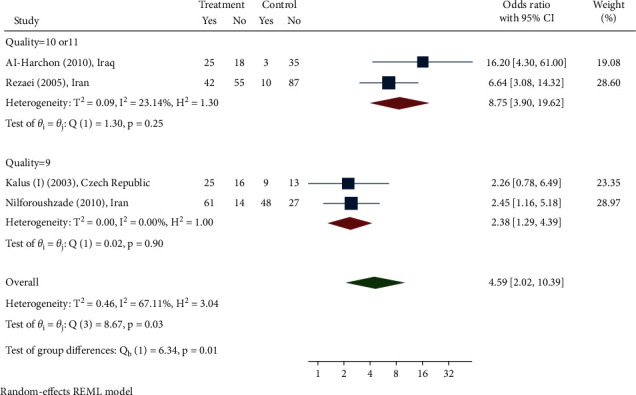
Odds ratio for *N. sativa's* ability to treat skin diseases.

**Figure 3:**
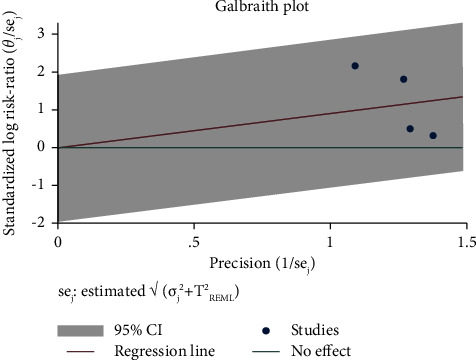
Galbraith plot in study on *N. sativa's* impact on skin conditions.

A total of 1159 patients were included in the systematic review. The mean SD age of the patients was 28.86 (4.49); [range: 18.3–51.4], with females accounting for 69% (506 out of 732) of the total. The follow-up mean SD was 8.16 (1.3) (ranged: 4 days to 24 weeks). The odds ratio (OR) was found to be 4.59 in a meta-analysis (95% CI: 2.02, 10.39). Whereas the null hypothesis in this systematic review was that lotion had no impact, OR 4.59 indicated that lotion could be effective. Based on the value of *I*2 = 67.11 (Figure 2) and Galbraith diagram (Figure 3), there was no significant heterogeneity between studies, but the value of *T*2 = 0.46 shows that there is significant heterogeneity within studies.

**Figure 4:**
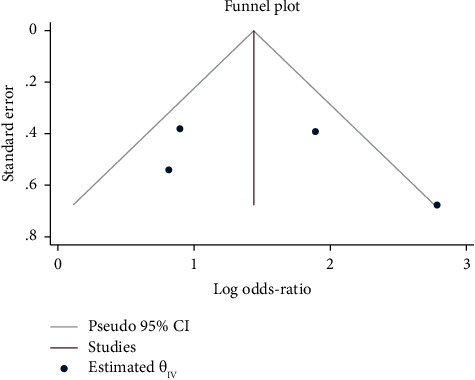
Funnel plot of publication bias for the effect of *N. sativa's* ability to treat skin diseases.

The results of the study do not appear to be impacted by publication bias, according to the funnel plot (Figure 4) and the Egger's test (*B* = 3.54, *p* value = 0.36), although the assessment of the publication bias is unreliable because there were only four papers included in the meta-analysis. Based on the findings of the sensitivity analysis, the results were influenced by one study [14]. When the recent study was taken out of the sensitivity analysis, the results were 3.45 less than the estimated value. As can be seen from the subgroup analysis, it appears that this study had an influence on the study's findings.

### 3.4. Quality Assessment of Included Studies

**Figure 5:**
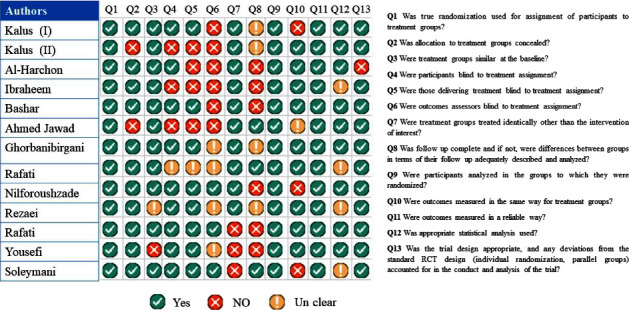
Quality assessment of study included in the systematic review the therapeutic effects of *N. sativa* on skin disease.

As a result, the quality of the included studies is assessed using the critical assessment tool for randomization clinical trials developed by the Joanna Briggs Institute. To evaluate the quality of case series and quasi-experimental research, please consult Figure 5.

## 4. Discussion

The current meta-analysis revealed that supplementation with *N. sativa* can potentially be effective in the treatment of different skin problems including atopic dermatitis, eczema, warts, keratosis, psoriasis, vitiligo, infant skin infections, and acne. However, the findings should be declared with caution because of heterogenicity. The studies included in the meta-analysis were homogeneous, and the differences between the studies did not significantly affect the estimated index, according to the value of the *I*2 index. However, there was heterogeneity within studies through using Galbraith diagram. Heterogeneity is an important consideration in systematic reviews, as high heterogeneity (more than 75%) indicates that it is not suitable to perform meta-analysis. To the best of our knowledge, there is no systematic review that has examined the effects of *N. sativa* on the improvement of symptoms of skin diseases. The study of the various forms of *N. sativa* showed that the oil supplement in topical form is more commonly reported. The pharmacological properties of *N. sativa* are more clearly observable in this form than in extract form because thymoquinone is a solvent in oil. A minimum of 4 weeks and a maximum of 24 weeks are recommended for the treatment period. The dermatological treatments of *N. sativa* are attributed to its strong antioxidant, anti-inflammatory, antimicrobial, and immunomodulatory potential, which altogether make it a promising skincare candidate. Since thymoquinone is one of the principal compounds of *N. sativa* and the concentration of it may be varied greatly depending on the storage and preparation of plant products, it is expected that the prescribed herbal products will be standardized according to the active ingredient thymoquinone. However, there is no information regarding the quantification or standardization of bioactive compounds among the clinical trials reviewed here. Standardization of herbal formulations is necessary in order to evaluate the quality of drugs based on the concentration of their active constituents or phytochemicals [24]. Standardization of herbal medicines carries an assurance of quality, efficacy, safety, and reproducibility [25]. Thymoquinone exists in tautomeric forms including the enol, keto, and mixtures in the oil of the plant. The keto form is responsible for the pharmacological features of thymoquinone [26]. Several potential mechanisms can be proposed for the observed ameliorating influences of *N. sativa* on infectious and noninfectious skin conditions including different types of allergies, autoimmunity, skin inflammations and wounds, and vitiligo. The findings of Ali and Meitei showed that the extract of *N. sativa*, as well as its active constituent thymoquinone, mimics the action of acetylcholine in melanin dispersion leading to skin darkening via stimulation of cholinergic receptors of a muscarinic nature within the melanophores of wall lizard. This study opens new vistas for the use of *N. sativa* active ingredient, thymoquinone, as a novel melanogen for its clinical application in skin disorders such as hypopigmentation or vitiligo [9].

Generally, there is an agreement regarding the impressive effects of *N. sativa* on inflammatory, oxidative, reactive oxygen species, and immunologic parameters in animal models. Houghton et al. showed that the anti-inflammatory action of TQ resulted from the prevention of eicosanoids generation, such as thromboxane B2 and leukotriene (LT) B4, by inhibiting both cyclooxygenase and 5-lipoxygenase, and in part via nonenzymatic peroxidation of membrane lipids [27]. TQ induce a significant inhibition on LTC4 synthase activity [28]. Velagapudi et al. demonstrated that TQ treatment elevated the activation of the NrF2/ARE pathway leading to the suppression on NF-*κ*B and following neuroinflammatory responses in microglia cells [29]. TQ was recently discovered to attenuate atopic dermatitis by reducing the levels of inflammatory cytokines, such as IL-4, IL-5, and IFN-gamma, and immunomodulatory cells in the blood. However, Liang et al. indicated that a high dose of TQ (higher than 16 *μ*M) possibly showed cytotoxicity on keratinocytes [8]. So, in clinical trials must be consider the standardization of the plant. 12 studies out of 14 in this review have reported the efficacy of essential oils of black cumin in skin disease. In light of the relatively low amount of TQ in the *N. sativa* essential oil, it seems that the skin healing effects of *N. sativa* are related to terpenoid compounds in addition to TQ. Therefore, the determination of these active constituents is recommended to achieve the *N. sativa* oil optimal dose to improve its efficacy. The previous investigations have shown the essential oil immunostimulatory effects on T cells and meaningfully inhibited allergy-associated cytokines IL-4 and IL-13 [30]. The antioxidant and anti-inflammatory effects of some other constituents of *N. sativa* essential oil such as p-cymene, t-anethole, thymol, carvone, *α*-terpineol, longifolene, and *β*-caryophyllene have been demonstrated in various studies [30–36].

## 5. Limitations

There were some limitations on these clinical studies, including the lack of reporting of any herbal standardization, the lack of measurement of chemical constituents of the plant, and study quality. The findings of this review should be considered cautiously due to various limitations. The fact that this study looked at skin disorders in general, and the number of clinical studies included in the meta-analysis was small (*n* = 4), so in the main analysis, therefore, limiting the sample size decreases the study's confidence level and increases the margin of error. The protocol for this review was has not been preregistered with PROSPERO, so it is a limitation of this review.

## 6. Conclusions

The efficacy of *N. sativa* essential oil and extract has been demonstrated in most clinical studies. This is the first systematic review assessing the available literature on the effects of *N. sativa* on skin diseases in clinical studies. In this systematic review article, we tried to give persuasive clues on the efficacy of *N. sativa* in skin disorders management and its mechanisms of action. However, more research is needed to completely evaluate and validate the efficacy or inadequacy of therapy with *N. sativa*, although it appears that it can be used as an alternative treatment to help people cope with skin problems.

## Figures and Tables

**Table 1:** The list of included studies on skin disease therapeutic effects with *N. sativa*.

Authors/years	Study design	Type of skin disease	Age	Sample size	Forms of drug use	Dosage	Duration	Improvement frequency	Clinical score index before/after treatment	References
Stern et al./2002	Prospective	Atopic dermatitis	Nr	20	Topical (ointment)	15% black seed oil other daily	4 weeks	Nr	1.71/1.01%	[10]
Kalus et al./2003	Double-blinded RCT	Atopic eczema	6–19	63	Oral (500 mg capsule)	Seed oil (40 mg/kg) three times a day	8 weeks	Invention group = 25/41, control = 9/22	Nr	[11]
Rezaei et al./2005	Double-blinded RCT	Wart	12–18	291	Topical (ointment)	30 g crude extract twice daily	6 weeks	Invention group = 42/43, control = 10/20	Nr	[12]
Nawab et al./2008	Before and after	Eczema	10–70	30	Topical (lotion)	25 mg oil 4 times a day	6 weeks	Nr	Eczema severity (itching) = 30/9 (papules) 19/4	[13]
Al-Harchon/2010	Single-blind RCT	Acne vulgaris	13–23	93	Topical (lotion)	10% oil twice daily	8 weeks	Invention group = 25/47, control = 3/38	Nr	[14]
Nilforoushzadeh et al./2010	Double-blinded RCT	Acute cutaneous leshmaniasis	20/81 ± 12/26	150	Topical (lotion)	60% oil twice daily	12 weeks	Invention group = 61/75, control = 48/75	Nr	[15]
Yousefi et al./2013	Double-blinded RCT	Eczema	18–60	60	Topical (lotion)	1 g seed oil twice daily	4 weeks	Nr	Nr	[16]
Bashar et al./2014	Double-blind, RCT	Arsenical keratosis	20–36	36	Oral (500 mg)	Seed oil	8 weeks	Nr	Palmar arsenical keratosis: 99.3 ± 21.5 and 62.3 ± 14.3	[17]
Ahmed Jawad et al./2014	RCT	Psoriasis	50–70	60	Topical (ointment), oral	Crude extract (10% (w/w) and 500 mg capsule) three times daily	12 weeks	Nr	PASI (psoriasis area and severity index) score in group 1: 9.0 ± 3.7/4.3 ± 2.0, group 2: 9.9 ± 3.4/5.4 ± 2.7, Group 3: 10.9 ± 2.7/4.2 ± 1.7	[18]
Ghorbanibirgani et al./2014	Double blind, RCT	Vitiligo	43.65 ± 3.21	52	Topical (lotion)	100 g seed oil	24 weeks	Nr	Vitiligo area scoring index in control group: 4.98 ± 4.81/4.62 ± 4.36, in invention 4.98 ± 4.81/3.75 ± 3.91	[19]
Rafati et al./2014	RCT	Infant skin infections	6–11 days	60	Topical (lotion)	33% oil three times a day	4 days	Nr	Nr	[20]
Rafati et al./2019	Double-blinded, RCT	Acute radiation dermatitis	≥18 years	62	Topical (gel lotion)	50 g gel lotion 5% twice a day	6 weeks	Nr	Nr	[21]
Sarac et al./2019	Clinical trials with a pre- and a post-treatment	Vitiligo	20–85	33	Topical (cream)	Oil/twice a day	24 weeks	Invention group = 23/33	Nr	[22]
Soleymani et al./2020	Double-blind RCT	Acne	14–35	60	Topical (gel lotion)	1% oil/twice a day	8 weeks	Nr	Comedone number invention = 8.07 ± 6.142/1.11 ± 1.812, in control + 8.87 ± 5.526/8.44 ± 5.437, papule number invention: 11.47 ± 6.426/1.89 ± 1.729, in control = 8.43 ± 4.116/7.31 ± 4.306	[23]

Nr: not reported.

## Data Availability

The data that supports the findings of this study are available in this article from the corresponding author upon request.

## Supplementary Materials

Supplementary Materials Supplementary file S2: critical appraisal for quasi-experimental studies included in the review.
