# Supplementary material for: The Therapeutic Effects of Nigella sativa on Skin Disease: A Systematic Review and Meta-Analysis of Randomized Controlled Trials
Source: Evid Based Complement Alternat Med. 2022 Dec 5;2022:7993579. doi: 10.1155/2022/7993579 (PMC9744621; doi:10.1155/2022/7993579)
Supplement: Supplementary Materials — Supplementary file S2: critical appraisal for quasi-experimental studies included in the review. [file 7993579.f1.zip › Supplementary File S1.docx]

Supplementary File S1: Critical appraisal for case-series studies included in the review

| **First Author (Ref)** | **1** | **2** | **3** | **4** | **5** | **6** | **7** | **8** | **9** | **10** | **Total Score** |
| --- | --- | --- | --- | --- | --- | --- | --- | --- | --- | --- | --- |
| **Gaudin** | Yes | Yes | No | Yes | No | Yes | No | Yes | No | No | 5/10 |

1. Were there clear criteria for inclusion in the case series?
2. Was the condition measured in a standard, reliable way for all participants included in the case series?
3. Were valid methods used for identification of the condition for all participants included in the case series?
4. Did the case series have consecutive inclusion of participants?
5. Did the case series have complete inclusion of participants?
6. Was there clear reporting of the demographics of the participants in the study?
7. Was there clear reporting of clinical information of the participants?
8. Were the outcomes or follow up results of cases clearly reported?
9. Was there clear reporting of the presenting site(s)/clinic(s) demographic information?
10. Was statistical analysis appropriate?

Supplementary File S2: Critical appraisal for quasi experimental studies included in the review

| **First Author (Ref)** | **1** | **2** | **3** | **4** | **5** | **6** | **7** | **8** | **9** | **Total Score** |
| --- | --- | --- | --- | --- | --- | --- | --- | --- | --- | --- |
| **Stern** | Yes | Yes | No | Yes | Yes | No | Yes | Yes | Yes | 7/9 |
| **Sarac** | Yes | Yes | No | No | Yes | No | Yes | Yes | No | 5/9 |
| **Nawab** | Yes | Yes | No | No | Yes | No | Yes | Yes | no | 5/9 |

1. Is it clear in the study what is the ‘cause’ and what is the ‘effect’ (i.e. there is no confusion about which variable comes first)?
2. Were the participants included in any comparisons similar?
3. Were the participants included in any comparisons receiving similar treatment/care, other than the exposure or intervention of interest?
4. Was there a control group?
5. Were there multiple measurements of the outcome both pre and post the intervention/exposure?
6. Was follow up complete and if not, were differences between groups in terms of their follow up adequately described and analyzed?
7. Were the outcomes of participants included in any comparisons measured in the same way?
8. Were outcomes measured in a reliable way?
9. Was appropriate statistical analysis used?
